# OD19 Future Health And Economic Burden Of Cardiovascular Disease In Type 2 Diabetes In Qatar, From 2023 To 2032

**DOI:** 10.1017/S0266462324001533

**Published:** 2025-01-07

**Authors:** Dina Abushanab, Daoud Al-Badriyeh, Clara Marquina, Danny Liew, Manal Al-Zaidan, Mohammed Ghaith Al-Kuwari, Jazeel Abdulmajeed, Zanfina Ademi

## Abstract

**Introduction:**

The Middle East and North Africa region accounts for the largest prevalence of type 2 diabetes (T2D). Qatar ranks top 10 for global T2D prevalence, with 17 percent in 2022. Also, 50 to 70 percent of the cardiovascular events occurred in T2D. We sought to estimate the future health and economic burden of cardiovascular disease (CVD) in T2D from 2023 to 2032.

**Methods:**

A dynamic multistate model in people with T2D was constructed. The demographic profile of the population was based on Qatari citizens and residents with T2D aged 40 to 90 years in 2022. First CVD events (i.e., myocardial infarction [MI] and stroke) were calculated via the 2013 Pooled Cohort Equation using data from Primary Health Care Corporation. Recurrent CVD events were sourced from the global Reduction of Atherothrombosis for Continued Health (REACH) registry. Outcomes were MI and stroke, years of life lived, quality-adjusted life years (QALYs), total direct and productivity loss costs. Utility and cost model inputs were drawn from published sources. The model adopted a Qatari societal perspective.

**Results:**

The model estimates 123,524 non-fatal MIs (95% uncertainty interval [UI]: 116,923, 130,065), 70,466 non-fatal strokes (95% UI: 67,945, 73,476) and 15,410 CVD deaths (95% UI: 15,217, 15,794), respectively. T2D population accrued 4,834,146 (95% UI: 4,781,235, 4,881,695) total years of life lived and 3,817,246 (95% UI: 3,756,963, 3,870,616) total QALYs. Direct costs accounted for 59.52 percent of the total costs, with a projection of QAR43.59 billion (USD11.98 billion) (95% UI: QAR9.14 billion [USD2.5 billion], QAR134.20 billion [USD36.87 billion]), while the total indirect costs were expected to exceed QAR29.65 billion (USD8.14 billion) (95% UI: QAR2.40 billion [USD659.34 million], QAR113 billion [USD31.04 billion]).

**Conclusions:**

This study highlights that the considerable rising burden of CVD in T2D in Qatar will impact not only the healthcare system but also the society overall. The findings may be used to prioritize strategies targeting T2D to prevent CVD burden.

